# Bionic 3D Path Planning for Plant Protection UAVs Based on Swarm Intelligence Algorithms and Krill Swarm Behavior

**DOI:** 10.3390/biomimetics9060353

**Published:** 2024-06-13

**Authors:** Nuo Xu, Haochen Zhu, Jiyu Sun

**Affiliations:** Key Laboratory of Bionic Engineering (Ministry of Education, China), Jilin University, Changchun 130022, China; xunuo21@mail.jlu.edu.cn (N.X.); zhuhc22@mails.jlu.edu.cn (H.Z.)

**Keywords:** plant protection UAV, path planning, swarm intelligence algorithm, bionic algorithm

## Abstract

The protection of plants in mountainous and hilly areas differs from that in plain areas due to the complex terrain, which divides the work plot into many narrow plots. When designing the path planning method for plant protection UAVs, it is important to consider the generality in different working environments. To address issues such as poor path optimization, long operation time, and excessive iterations required by traditional swarm intelligence algorithms, this paper proposes a bionic three-dimensional path planning algorithm for plant protection UAVs. This algorithm aims to plan safe and optimal flight paths between work plots obstructed by multiple obstacle areas. Inspired by krill group behavior and based on group intelligence algorithm theory, the bionic three-dimensional path planning algorithm consists of three states: “foraging behavior”, “avoiding enemy behavior”, and “cruising behavior”. The current position information of the UAV in the working environment is used to switch between these states, and the optimal path is found after several iterations, which realizes the adaptive global and local convergence of the track planning, and improves the convergence speed and accuracy of the algorithm. The optimal flight path is obtained by smoothing using a third-order B-spline curve. Three sets of comparative simulation experiments are designed to verify the performance of this proposed algorithm. The results show that the bionic swarm intelligence algorithm based on krill swarm behavior reduces the path length by 1.1~17.5%, the operation time by 27.56~75.15%, the path energy consumption by 13.91~27.35%, and the number of iterations by 46~75% compared with the existing algorithms. The proposed algorithm can shorten the distance of the planned path more effectively, improve the real-time performance, and reduce the energy consumption.

## 1. Introduction

Plant protection UAVs have the characteristics of small size, light weight, and easy operation and have wide application prospects in pesticide spraying [1], crop seeding [2], soil environmental monitoring [3], treatment of diseases and pests [4], and other fields [5,6]. The use of plant protection UAVs has a variety of advantages and obvious control effects; specifically, UAVs can quickly address sudden diseases and pests, carry various types of agents, and reduce the degree of damage to farmland and crops. Before the plant protection operation, it is necessary to plan the operation trajectory of the plant protection UAV in advance. The shape of farmland in plain areas is relatively regular, and full-coverage path planning under fixed-altitude flight is usually adopted for plant protection operations [7]. In mountainous and hilly areas, the terrain is changeable, the farmland plots are not directly connected to each other, and most of the plots are small and irregular. When the plant protection UAV completes its operation in one plot, it needs to fly to the next plot for the next plant protection operation. Due to the complex terrain, it is very difficult to implement flying with a fixed altitude or operator remote control. To ensure that the plant protection UAV can travel smoothly between the operating plots and ensure the optimal flight path during operation, it is necessary to study the path planning for plant protection UAVs in complex 3D environments.

Existing path planning algorithms are mainly divided into two categories: traditional graph search algorithms and heuristic intelligent algorithms [8,9]. The main feature of traditional graph search algorithms is path searching after map rasterization, which determines the shortest path and optimal efficiency [10]. Typical graph search algorithms include Dijkstra’s algorithm [11], the A-star algorithm [12], and the artificial potential field algorithm [13]. Traditional map search algorithms have the advantages of high calculation accuracy and good real-time performance but poor path continuity and unreliable search performance in high-dimensional space. Therefore, traditional map search algorithms are not suitable for plant protection UAV operations in complex terrain areas. The heuristic intelligent algorithm, also known as the swarm intelligence algorithm, solves the path planning problem for UAVs by imitating the natural behavior of animals (birds, ants, and bees) [14,15,16]. Compared with traditional graph search algorithms, swarm intelligence algorithms can handle incomplete information in complex environments, easily ensure path continuity, and are more suitable for path planning in 3D space. Common swarm intelligence algorithms include the ant colony algorithm [17], particle swarm optimization algorithm [18], and artificial bee colony algorithm [19]. Although these swarm intelligence algorithms can be used to generate the flight path of plant protection UAVs in 3D space, they have many disadvantages, such as uncertain convergence speed and long planning time, and they are not suitable for application scenarios with high real-time requirements.

Recently, many scholars have been contributing to a growing body of research on swarm intelligence algorithms and their application to UAV flight path planning. Yang et al. [20] improved the light intensity pre-processing and calculation method based on the Plant Growth Algorithm (PGPP), resulting in a smoother planned path for UAS with their proposed Light-Sensitive Enhanced Plant Growth Algorithm (PEPG). Jiang et al. [21] introduced a novel Golden Jackal Optimization Algorithm (SCMGJO) by combining the sine-cosine algorithm with the Cauchy mutation algorithm, enhancing the global exploration capability of UAVs, and improving the efficiency of path planning algorithms to obtain the optimal solutions. Chen et al. [22] combined the Gray Wolf algorithm (GWO) with the artificial potential field method (APF) to propose a Fusion Optimization Algorithm (GGO-APF), which enhanced the stability, safety, and efficiency of UAVs’ path planning operations. Machmudah et al. [23] utilized Particle Swarm Optimization (PSO) to optimize fixed-wing UAVs’ flight constraints, reducing the load coefficient during inclined turning mechanisms within allowable speed ranges. Liu et al.’s study [24] proposed an improved Sparrow Search Algorithm based on multiple strategies using the Cauchy reverse learning and sine-cosine algorithm to optimize the UAV path planning parameters such as length, turn time, and execution time. Although the proposed improved method optimizes the traditional swarm intelligence algorithm, it also has many defects, including the high complexity of the algorithm, which cannot be applied in multiple obstacle environments. Therefore, there is a need for further research into swarm intelligent routing algorithms that offer better performance specifically tailored for plant protection UAV operations.

There are many animal cluster phenomena in nature, and animals can exhibit effective movement behavior in the living environment. Antarctic krill is a species of krill living in Antarctica. An adult krill’s body length is approximately 6 cm, their weight is approximately 2 g, their average lifespan is approximately 6 years, their main food is plankton, and they are a very important species in the Antarctic ecosystem. Antarctic krill generally live in colonies, and this feature is considered one of the factors that contribute to the reproductive success of their species [25]. Researchers have conducted many studies on krill populations in laboratory and field environments to understand the structure and function of krill populations [26,27,28], focusing on the movement mode of krill populations in 3D marine environments, their ability to track nutrients in the water, flexible avoidance of enemy hazards, and efficient long-distance swimming [29]. The main characteristics of the krill swarm movement strategy are that it is simple and efficient and can successfully complete path planning in a 3D water space with a simple and effective method. This processing efficiency provides inspiration for the design of the path planning algorithm for plant protection UAVs.

In this paper, we propose a bionic 3D path planning scheme to be applied to plant protection UAVs that can be used to plan the flight path of plant protection UAVs in a complex 3D environment. The main contributions of this paper are summarized as follows:

- Based on the swarm intelligence algorithm and inspired by krill swarm motion patterns, a bionic swarm intelligence algorithm suitable for three-dimensional environment is proposed. The algorithm changes the planning process at any time according to the current state of the UAV, and can connect different planning processes to adapt to the different working environments.
- Make the plant protection UAV traverse the obstacle environment without collisions occurring under various complex terrain conditions.
- Smooth flight path trajectories can be generated by using third-order B-spline curves to connect path points in three-dimensional space smoothly.
- Through three sets of simulation experiments, compared with the two commonly used swarm intelligence algorithms, the proposed algorithm reduces the length of UAV flight paths, the algorithm operation time, the flight energy consumption, and the number of iterations.

The rest of this paper is organized as follows: the second chapter introduces the path planning problem description for the plant protection UAV and krill swarm movement strategy; the third chapter proposes the bionic 3D path planning and design method; the fourth chapter verifies the algorithm’s performance through three groups of comparative experiments; and the fifth chapter summarizes our research and proposes future research directions.

## 2. Bionic Path Planning Inspired by Biological Behavior

To better realize the flight path planning for plant protection UAVs and inspired by the ability of living organisms to adopt different living modes and movement modes under different external conditions, a better path planning effect can be achieved by applying it to the obstacle avoidance and shuttle operations by plant protection UAVs over complex terrain.

### 2.1. Path Planning Problem Description for Plant Protection UAVs

Plant protection UAVs usually operate in small plots in mountainous and hilly areas, so they cannot fly directly to the next cultivated area after completing their operations in the first area like they can during the process of plant protection in plains areas. Instead, the UAV needs to make multiple flight transfers during the transition process, and due to the uneven relief and complex terrain in mountainous and hilly areas, plant protection UAVs need to plan a safe and reliable 3D flight path with a short flight distance and less time.

**Figure 1:**
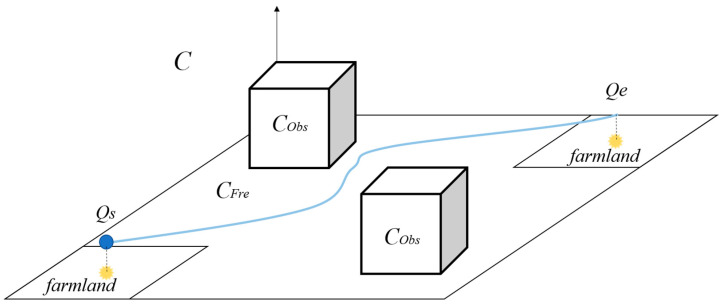
Path planning spatial relationship.

During the path planning for plant protection UAVs, the surrounding terrain environment information needs to be constrained. In mountainous and hilly areas, the obstacles mainly include growing trees and irregular terrain height. To ensure that the plant protection UAV does not collide with obstacles during flight, it is stipulated that the flight height *H* should be above the ground height *H_v_* of the UAV fuselage, and the horizontal distance between it and the obstacles around the UAV should be above *H_l_*. The range is an impassable area. The whole working space of the plant protection UAV is *C*, and the impassable area *C_Obs_*, which is composed of the base information, is the terrain constraint of the path planning of the plant protection UAV. *C_Fre_ = C ∩ C_Obs_* is the free flying movement space of the plant protection UAV. In 3D space, the position of the plant protection UAV can be represented by a 3D vector *Q_i_* = {*x_i_*, *y_i_*, *z_i_*}, and the UAV path planning problem can be transformed into a collection of all path points in the UAV flight path consisting of vector *Q_i_* with the smallest length from the starting point *Q_s_* to the ending point *Q_e_* in free-flying motion space *C_Fre_*. The spatial relationship of the path-planning tasks is shown in Figure 1.

### 2.2. Krill Cluster Biological Behavior

We chose krill living in the marine environment as the bionic prototype since they can move in various directions in the water body, which is very similar to the task of path planning in the workspace of plant protection UAVs. The movement behavior pattern of krill clusters in marine living environments was observed and applied to the path planning of plant protection UAVs.

**Figure 2:**
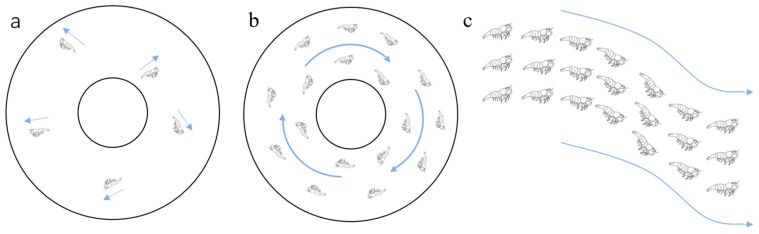
Krill biological behavior. (**a**)When there is a small amount of krill, the movement behavior of krill is random jumping and irregular, (**b**)as the number increases, the movement mode of krill becomes collective, and (**c**) when the number more, the speed direction of krill shows a pattern of individuals moving towards the front and below, while moving away from the front and back. The blue arrow represents the direction of movement.

Krill is a kind of miniature crustacean living in Antarctica, and it generally has two living modes, solitary and social [25]. When the population density is low, krill are in a solitary state. When the population density reaches a certain threshold, there is a so-called “phase transition” into a gregarious mode. When there is only a small number of krill, the motion behavior of krill is random and involves hopping, as shown in Figure 2a. As their numbers increase, krill movement becomes more collective, as shown in Figure 2b. The movement regularity of krill has a strong relationship with the population density; that is, it exhibits random movement at a low density and homotropic movement at a high density.

The clustering law of krill groups is very different from that of general marine cluster organisms. The swimming speed of krill groups is adjusted according to the speed of the individuals in front of them, but the direction of advance is adjusted according to other individuals in the vertical dimension; in addition, their speed and direction are closer to those of individuals in front and below but far away from individuals in the front and rear [28], as shown in Figure 2c. The main reason why krill use more vertical dimension information is that the krill’s eyes are on the top of their heads and can only look up, not down, which creates partial visual blindness below and horizontally. Second, when krill are frightened, their abdomens glow, a message that can be transmitted to other individuals. These are two main reasons why the krill’s visual focus is mainly on the vertical dimension.

### 2.3. Bionic Path Planning Strategies Inspired by Krill Clusters

By simulating the biological movement strategy of krill in the case of a swarm, it can be applied to path planning for plant protection UAVs in 3D space. The behavior patterns of krill in different situations are analyzed. When krill are in a single individual state, random and irregular motion modes are adopted, and the motion mode of krill is highly uncertain. When a krill is in a cluster state, the isotropic motion mode is adopted, and the motion mode of the krill has a unified regularity. The krill’s clustering behavior generally exhibits the following three characteristics: collective foraging activities, finding and avoiding enemy predation, and moving to new gathering places along with ocean currents. These are similar to the problems faced by plant protection drones in field work. In terms of UAV path planning, the planning process can be changed at any time according to the current state of the plant protection UAVs, and different planning processes can be connected to each other to adapt to different workplaces.

In a space, each drone is assumed to be a biological unit, and the place with the largest number of biological units is the place with the most nutrients in the film. Based on this feature, the foraging, gathering, and cruising behaviors of the biological unit groups are imitated to find the optimal path for the drone to the target location.

**Figure 3:**
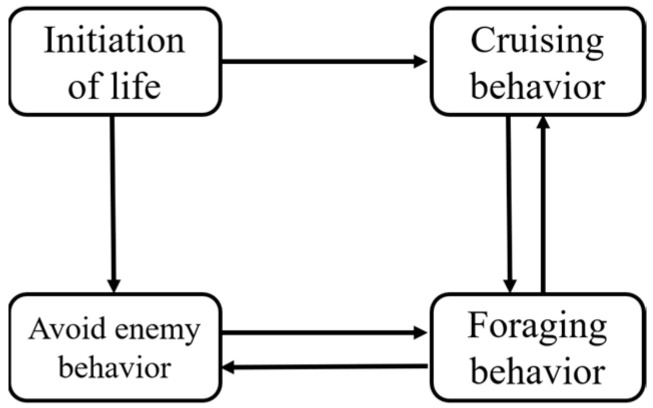
Life activity diagram.

The life activities of biological units are divided into three kinds of biological behaviors: foraging behavior, enemy avoidance behavior, and cruising behavior. These are shown in Figure 3. Foraging behavior is defined as a behavior in which biological units advance in the direction of food abundance. In the search for the optimal path, foraging behavior iterates in the direction of the global optimum, similar to the visual concept in the fish swarm model. Enemy avoidance behavior is defined by two rules for each biological unit: one is to move to the center of neighboring biological units as far as possible; the other is to avoid overcrowding among individuals to realize the biological units’ clustering ability. Cruising behavior is defined as the behavior of chasing the nearest biological unit with the highest fitness. In the search for the optimal path, it is the process of advancing to the nearby individual with the optimal state.

Suppose that in a 3D target search space, there are *N* biological units that form a biological population, and the individual state of each biological unit can be expressed by the vector *X* = (*x*_1_, *x*_2_, …, *x*_n_), where *x_i_*(*i* = 1, …, *n*) is the variable for finding the optimal path and is also each control point in path planning. The food concentration at the current location of the biological unit is expressed as *Y = f*(*X*), where *f*(*X*) is the objective function, and each biological unit has a corresponding fitness. The distance between the individuals of a biological unit is d = ||x_i_ − x_j_||, *U* is the perceptual range of a biological unit, *step* is the moving step of a biological unit, *δ* is the crowding factor of a biological unit, and *T* is the maximum number of foraging attempts by a biological unit.

## 3. Bionic 3D Path Planning Implementation

### 3.1. Swarm Intelligence Algorithms

#### 3.1.1. Ant Colony Algorithm

**Figure 4:**
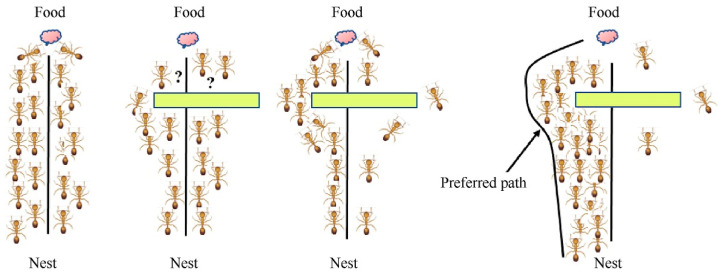
Life activity diagram. “?” presents the two ants are not clear about their current positions and cannot determine their path forward.

The ant colony algorithm simulates the foraging behavior of ants in nature [30]. When looking for food sources, ants release pheromones along their path and perceive the pheromones released by other ants at the same time. The pheromone concentration represents the path distance, and the higher the pheromone concentration, the shorter the corresponding path distance. Ants preferentially choose the path with a higher pheromone concentration with a higher probability and release a certain amount of pheromone to enhance the pheromone concentration on the path, which forms a positive feedback loop; eventually, the following ants can find an optimal path from the nest to the food source, as shown in Figure 4 [31].

The number of the whole group of ants is set to *m*, the number of food items is set to *n*, the distance between food items is set to *n_i_* and *n_j_* for *d_i_*(*i*,*j* = 1, 2, …, *n*), and the *t* time food *n_i_* and *n_j_* connection path pheromone concentration is set to *τ_ij_* (*t*).

Ant *k*(*k* = 1, 2, …, *n*) chooses the next food to visit according to the pheromone concentration on each food connection path. Let *P_ij_*(*t*) represent the probability that ant *k* transfers from food *i* to food *j* at time *t*, and its calculation formula is expressed in Equation (1) as follows:

(1) $$P_{ij}=\left\{\begin{array}{cc} \frac{{\left[\tau_{ij}\left(t\right)\right]}^{\infty }\times {\left[\eta_{ij}\left(t\right)\right]}^{\beta }}{\sum_{{allow}_{k}}{\left[\zeta_{is}\left(t\right)\right]}^{\infty }\times {\left[\eta_{is}\left(t\right)\right]}^{\beta }} & ,\;s\in {allow}_{k}\\ 0 & ,\;s\notin \;{allow}_{k} \end{array}\right.$$

where *η_ij_*(*t*) is the heuristic function, *η_ij_*(*t*) = $1/d_{ij}$ represents the expected degree of the ant transferring from food *i* to food *j*, and *allow*_k_ (*k* = 1, 2, …, *n*) is the set of food to be accessed by ant *k*. *allow*_k_ starts with (*n* − 1) elements in *allow*_k_. As time goes on, *allow*_k_ gradually decreases until it is empty. *α* is the important pheromone degree factor, and the larger its value, the greater the role of the pheromone concentration in metastasis. *β* is the important degree factor of the heuristic function, and the larger its value, the greater the role of the heuristic function during transfer.

When the ant releases a pheromone, the pheromone on each food connection path gradually disappears. Parameter *ρ*(0 < *ρ* < 1) is set to indicate the degree of pheromone play. When all ants complete a cycle, the pheromone concentration on each food connection path needs to be updated in real time. The specific formula is expressed in Equation (2) as follows:

(2) $$\left\{\begin{array}{c} \tau_{ij}\left(t+1\right)=\left(1-\rho \right)\times \tau_{ij}\left(t\right)+{∆\tau }_{ij}\\ {∆\tau }_{ij}=\sum_{k=1}^{m}{∆\tau }_{ij}^{k} \end{array},\;\rho (0<\rho <1)\right.$$

where ${∆\tau }_{ij}^{k}$ represents the concentration of pheromone released by *k* ants on the connection path between food *i* and food *j*, and ${∆\tau }_{ij}$ represents the sum of the concentration of pheromone released by all ants on the connection path between food *i* and food *j*.

#### 3.1.2. Artificial Colony Algorithm

The artificial bee colony algorithm simulates the honey-gathering behavior of bees in nature [32]. Bees in nature can always find high-quality honey sources in any environment with high efficiency and can adapt to environmental changes. The honey collection system of a bee colony is mainly composed of a honey source, collecting bees, following bees, and detecting bees. A honey source is a feasible solution to the optimization problem, and it is the basic object to be addressed in the artificial bee colony algorithm. The quality of the honey source is evaluated by the fitness (objective function) of the actual problem. The greedy criterion [33] is adopted to compare the optimal solution in memory and the neighborhood search solution. When the search solution is superior to the optimal solution in memory, the solution in memory is replaced but remains unchanged when the opposite is true. After all of the foraging bees complete the neighborhood search, the foraging bees return to the hive and share nectar source information with the following bees. According to the foraging bees’ honey source information, the honey source is selected with a certain probability. The probability of foraging bees with a large honey quantity is higher than that of foraging bees with a small honey quantity. The follower bees search in the area adjacent to the nectar source, and the greedy criterion is used to compare the follower bees’ search solution with the original bees’ solution. When the search solution is better than the original bees’ solution, the original bees’ solution is replaced, and vice versa. If a honey source falls into the local optimum, scout bees are used to search for the optimal solution.

Randomly generated in the search space of the *S_n_* nectar source location, these locations also represent the first generation in the bees’ search for the optimal location. Bees search for new nectar sources near the nectar source, and the search formula is expressed in Equation (3) as follows:

(3) $$x_{ij}=x_{ij}+rand\times (x_{ij}-x_{kj})$$

where *j* represents a dimension of the solution, *i* represents the current forager, and *k* is the number of foragers other than *i*.

The gatherer bees return to the hive and share nectar source information with the follower bees. Each follower bee chooses a specific honey source with a certain probability according to the fitness of the honey source and gathers honey around it. If a honey source in a certain place cannot be used to find a better nearby honey source after several searches, then it is considered that it has fallen into local optimization. Then, the scout bees are invoked to randomly generate a new honey source location using a relatively time-consuming method, and the search formula is expressed in Equation (4) as follows,

(4) $$x_{ij}=x_{ij}+rand\times map(j)$$

The *map*(*j*) function is the return length of the search path of the *j*-th dimension in the map.

### 3.2. Bionic 3D Path Planning Algorithm Description

Aiming at the motion behavior of krill clusters in 3D space, a bionic path planning algorithm combined with a swarm intelligence algorithm is proposed to solve the path planning problem for UAVs. It consists of “foraging behavior”, “enemy avoidance behavior”, and “cruising behavior”.

#### 3.2.1. Foraging Behavior Description

**Figure 5:**
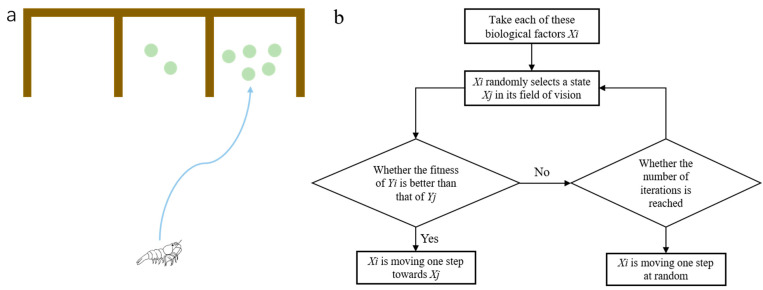
Foraging behavior. (**a**) A schematic diagram of foraging behavior; (**b**) The foraging proces diagram.

Foraging behavior refers to a behavior in which biological units swim along a path with plenty of food. The foraging behavior is shown in Figure 5a. Biological factor *X_i_* randomly selects a state *X_j_* in its field of vision, calculates their objective function values and makes a comparison. *X_j_* is calculated by Equation (5) as follows:

(5) $$X_{j}=X_{i}+rand()\times U$$

where *rand*() is a random number weight factor function of 0–1. If *Y_j_* is found to be better than *Y_i_* (*Y_j_* and *Y_i_* are fitness values of *X_j_* and *X_i_*, respectively), *X_i_* moves one step in the direction of *X_j_*; otherwise, *X_i_* continues to select state *X_j_* in its field of vision to determine whether the condition of advance is met. The movement calculation is specified in Equation (6), and the random movement calculation is expressed in Equation (7) as follows,

(6) $$X_{next}=X_{i}+rand()\times step\times \frac{X_{j}-X_{i}}{\left||X_{j}-X_{i}|\right|}$$

(7) $$X_{next}=X_{i}+rand()\times step$$

After T repeated attempts, if no solution satisfying the advancing condition is found, *X_i_* moves one step at random to reach a new state. The foraging behavior flow is shown in Figure 5b.

#### 3.2.2. Enemy Avoidance Behavior Description

**Figure 6:**
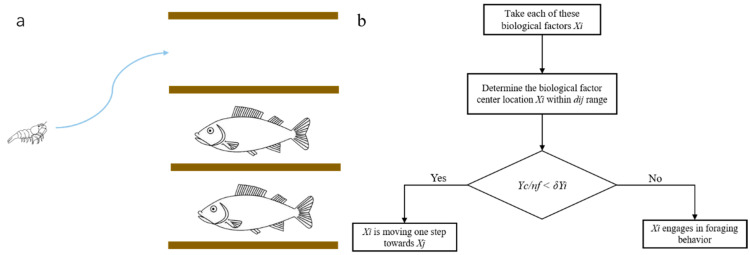
Enemy avoidance behavior. (**a**) Schematic diagram of enemy avoidance behavior; (**b**) Flow chart of enemy avoidance behavior.

Enemy avoidance behavior refers to the spontaneous gathering of biological factors to ensure their own survival and avoid harm in the process of movement. Enemy avoidance behavior is shown in Figure 6a. Biological factor *X_i_* searches the number *n_f_* of other biological factors in its field of vision (*d_ij_* < *U*) and calculates the central position *X_c_* of all biological factors. *X_c_* is calculated by Equation (8) as follows,

(8) $$X_{c}=\frac{X_{i}+X_{j}}{2}$$

If $Y_{c}/n_{f}<\delta Y_{i}$ (*Y_c_* and *Y_i_* are the fitness values of *X_c_* and *X_i_*, respectively) it indicates that the current central position of the biological factors is in a better state and not too crowded, and then *X_i_* moves one step toward the central position of the biological factors; otherwise, it indicates that the current central position of the biological factors is in a poor state and crowded. The foraging behavior and enemy avoidance behavior flows are shown in Figure 6b.

#### 3.2.3. Cruising Behavior Description

**Figure 7:**
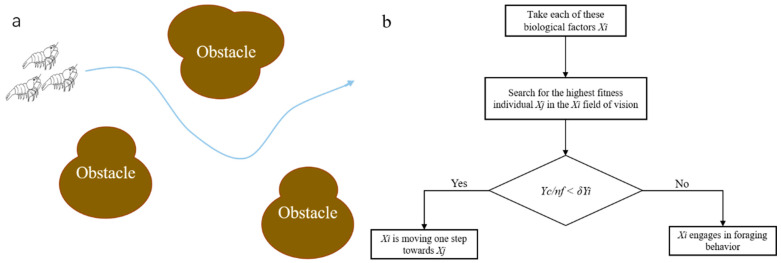
Cruising behavior. (**a**) A schematic diagram of parade behavior. (**b**) Tour behavior flowchart.

Cruising behavior refers to a behavior in which biological factors move in the optimal direction in their visual field. The cruising behavior is shown in Figure 7a. Biological factor *X_i_* searches for the individual *X_j_* with the highest fitness in its visual field (*d_ij_ < U*), whose fitness value is *Y_j_*, and explores the number of individuals *n_f_* in the visual field of biological factor *X_j_*. If $Y_{c}/n_{f}<\delta Y_{i}$, then the central position of *X_j_* is in a better state and not too crowded. Then, *X_i_* moves one step toward the position of *X_j_*; otherwise, foraging behavior is performed. The flow chart for cruising behavior is shown in Figure 7b.

#### 3.2.4. Overall Framework of the Bionic 3D Path Planning Algorithm

**Figure 8:**
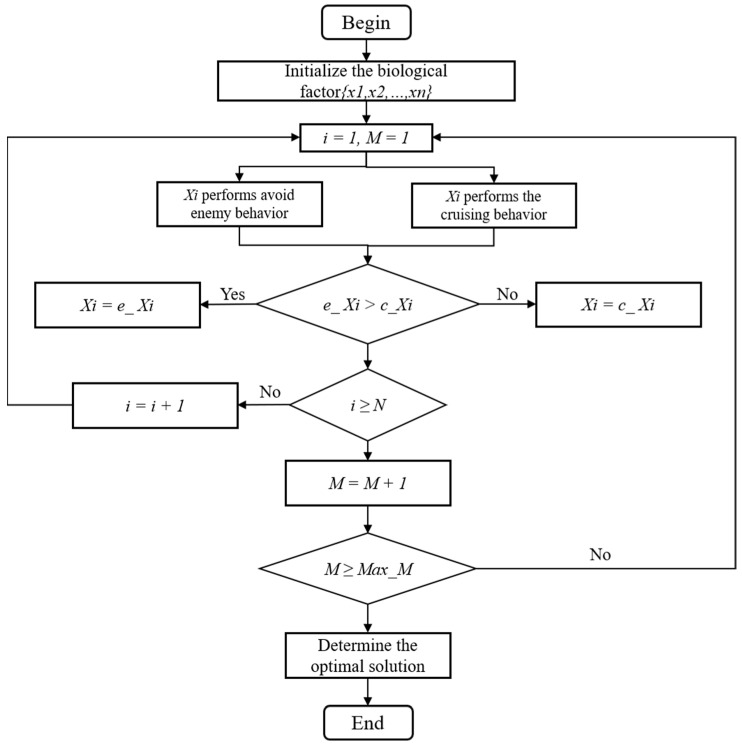
Algorithm general flow chart.

The overall flow diagram of the bionic 3D path planning algorithm is shown in Figure 8. During the operation process, the algorithm conducts both enemy avoidance behavior and cruising behavior and conducts foraging behavior when the judgment condition of Y/n<δY is not met. Foraging behavior belongs to the behavioral mode selected by the biological factors when the crowd is too large near the gathering object or the cruising object. In the process of foraging, if no biological factor with a higher fitness than its own is found in the current biological factors, it moves randomly according to the step size *step*. Finally, the fitness values obtained from the enemy avoidance behavior and cruising behavior are compared, and the biological factor with the better current state is selected as the calculation object for the next operation.

**Figure 9:**
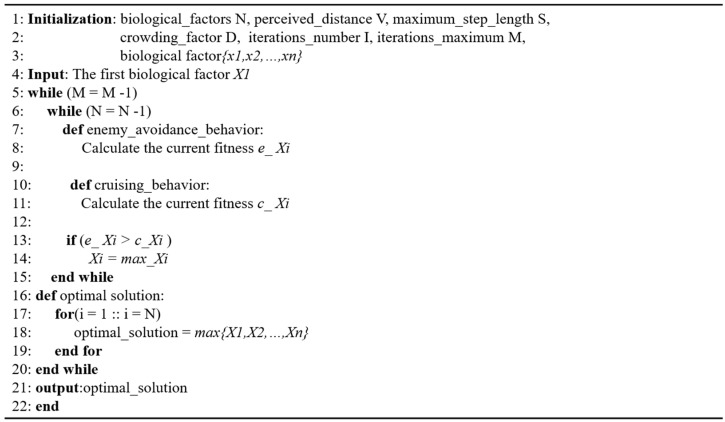
Algorithmic pseudocode.

The pseudocode of the bionic 3D path planning algorithm is shown in Figure 9. First, the basic parameters are set: the total number of biological factors *N*, perceptual distance *V*, maximum length of movement *S*, crowding factor *D*, iteration number *I*, maximum iteration number *M*, and biological factor population initialization {*X*_1_, *X*_2_, …, *X_n_*}. In the calculation for the first biological factor, the biological factor performs enemy avoidance behavior and cruising behavior. The two behaviors are independently calculated to obtain the position of the enemy avoidance behavior and the cruising behavior. The fitness of the corresponding position is obtained through the calculation of the position, the advantages and disadvantages of the two kinds of fitness are judged, and the corresponding position with better fitness is selected to calculate each biological factor repeatedly. When all the biological factors have been calculated, the iteration process is replicated. The process iterates *M* times, and the output result of the last iteration is the result of the optimal path.

### 3.3. Three-Dimensional Map Construction

The environmental information needed for 3D path planning needs to be extracted from the terrain model, and good terrain modeling can effectively improve the efficiency of path planning. The complex and changeable unknown terrain in the UAV flight environment is described by an exponential function, and the mathematical model can be expressed by Equation (9) as follows:

(9) $$z\left(x,y\right)=\sum_{i=1}^{n}h_{i}exp\left[-{\left(\frac{x-x_{i}}{x_{si}}\right)}^{2}-{\left(\frac{y-y_{i}}{y_{si}}\right)}^{2}\right]$$

where (*x_i_*, *y_i_*) is the central coordinate of the *i*th obstacle; *h_i_* is the terrain parameter; the control height variables *x_si_* and *y_si_* are the attenuation and control slope of the *i*th obstacle along the *x* and *y* axes, respectively; and *n* is the total number of obstacles.

### 3.4. Three-Dimensional Path Smoothing Processing

With a total of *n* control points {*P*_0_, *P*_1_, …, *P_n_*}, the control points are used to define the trend and limit range of the spline curve; then, the *k*-order B-spline curve is defined in Equation (10) as follows,

(10) $$P\left(u\right)=\left[P0\;\;P1\;\;\ldots \;\;Pn\right]\left[\begin{array}{c} B_{0,k}\left(u\right)\\ \begin{array}{c} B_{1,k}\left(u\right)\\ \begin{array}{c} \ldots \\ B_{n,k}\left(u\right) \end{array} \end{array} \end{array}\right]=\sum_{i=0}^{n}P_{i}B_{i,k}(u)$$

where *B_i_*_,*k*_(*u*) is the *i*-th *k*-order B-spline basis function corresponding to the control point *P_i_*, *u* is the independent variable, and the basis function has the following de Boor recursive formula, which is expressed in Equation (11) as follows,

(11) $$B_{i,k}\left(u\right)=\left\{\begin{array}{ll} \left\{\begin{array}{ll} 1, & u_{i}\leq \;u\leq \;u_{i+1}\\ 0, & u<u_{i},\;\;\;u>u_{i+1} \end{array}\right., & k=1\\ \frac{u-u_{i}}{u_{i+k-1}-u_{i}}B_{i,k-1}\left(u\right)+\frac{u-u_{i}}{u_{i+k-1}-u_{i}}B_{i+1,k-1}\left(u\right), & k\geq 2 \end{array}\right.$$

In the equation, *U* = {*u*_0_, *u*_1_, …, *u_n_*} is the node vector, and the size of each node has the property of monotonically increasing, that is, *u_i_ ≥ u_i+_*_1_. Therefore, it can be seen that the B-spline basis function can be determined if the number of iterations and node vector are well determined.

**Figure 10:**
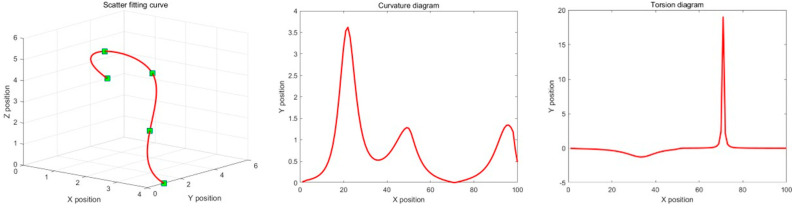
Third-order B-spline smoothing process. Green nodes present sampling points, the purpose is to smooth the processing path.

The working space of plant protection UAVs is a 3D environment; at this time, a third-order B-spline basis function is adopted to address scatter points. In 3D space, the curve is described by two indices of curvature and flexibility, and the curvature and flexibility are required to be continuous without mutation. The coordinates of the optimal waypoints X, Y, and Z for the plant protection UAV are regarded as function values of parameter t, and the range of parameter t is set to [0, 1]. They represent the first scatter point and the last scatter point, respectively. Using a third-order B-spline function, the smooth connection of 3D scatter points can be realized. Using {*P*0, *P*1, *P*2, *P*3, *P*4} as five example control points, their fitting curves, curvatures, and flexure graphs are shown in Figure 10.

### 3.5. Overall Path Planning Process

**Figure 11:**
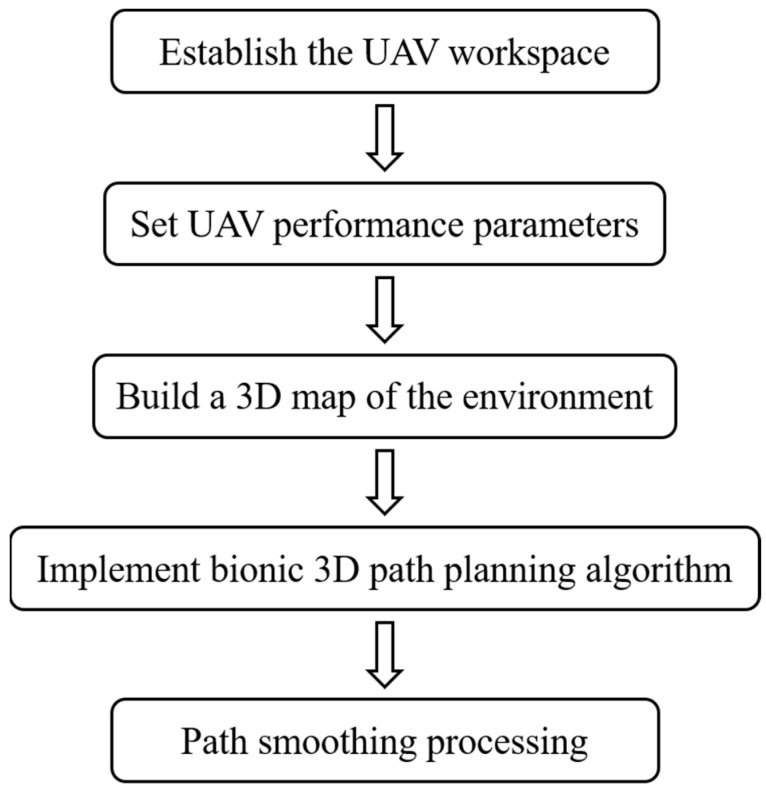
Third-order B-spline smoothing process.

The specific implementation process of path planning is shown in Figure 11 when the plant protection UAV transitions between operating fields. The plant protection drone establishes the working space and determines the starting and ending position of the farmland. The body performance parameters of the plant protection UAV are input, and maneuvering performance indicators such as the flight altitude, flight speed, altitude to the ground, distance to the surrounding obstacles, and flight energy consumption are set as constraints. A 3D map of the environment is constructed, and the working space, terrain constraint, and movement space of plant protection UAVs are established. The bionic 3D path planning algorithm is used to plan the path, and the optimal path coordinate point set is obtained. The optimal path is smoothed in the 3D environment to obtain the actual flight path of the plant protection UAV and complete the whole path planning task.

## 4. Experimental and Simulation Analysis

**Figure 12:**
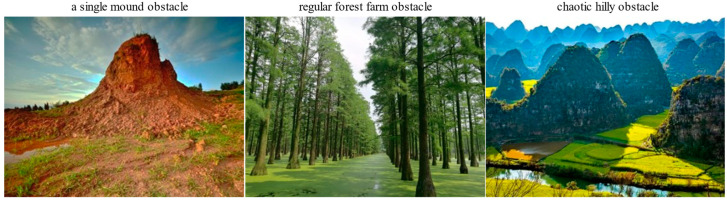
Real-world obstacle environments as depicted.

In order to validate the effectiveness of the bionic 3D path planning algorithm, we initially imposed constraints on the operational capabilities of the plant protection UAV. Three sets of simulation experiments were conducted under constrained conditions, simulating real-world obstacle environments as depicted in Figure 12. The first set focused on evaluating the algorithm’s performance in a single-mound obstacle environment, with comparisons made to the ant colony and artificial bee colony algorithms across four key metrics: path length, planning time, energy consumption, and iteration convergence. The second set aimed to assess the algorithm’s performance in a regular forest farm obstacle environment, again comparing it with the ant colony and artificial bee colony algorithms using similar metrics. Lastly, the third set targeted the validation of the algorithm’s performance in a chaotic hilly obstacle environment while considering comparable aspects against other algorithms. Three-dimensional map construction and comparison among these simulation experiments were carried out using MATLAB 2021a on a DELL notebook G15 5511 equipped with an Intel i7-11800H CPU, 16.0 GB RAM running Windows 10.

### 4.1. UAV Body Performance Constraints

In addition to the direct impact of terrain factors on the path planning of plant protection UAVs, it is also necessary to consider the objective impact of the body performance of plant protection UAVs on path planning to make the planned path feasible. The fuselage performance of the plant protection UAVs mainly includes the minimum height of the fuselage from the ground *H_v_*, the minimum horizontal distance of the fuselage from surrounding obstacles *H_l_*, and the flight energy consumption per unit distance *R*. The physical performance constraints are shown in Table 1.

The minimum height between the fuselage and the ground *H_v_* sets a minimum safe distance from the ground for the plant protection UAV to prevent working conflicts between the UAV and crops on the surface of the working space, which may cause additional economic losses. The minimum horizontal distance between the fuselage and the surrounding obstacles *H_l_* sets a buffer distance between the plant protection UAV and the surrounding obstacles to prevent collisions between the UAV and the surrounding obstacles from causing damage to the UAV hardware. The flight energy consumption per unit distance *R* refers to the power consumed per unit distance during horizontal or vertical flight by plant protection UAVs. Due to the influence of acceleration, the energy consumption of plant protection UAVs is different during horizontal and vertical flight.

The iteration duration can be provided by the program’s built-in function, and the formula for calculating the path length is expressed in Equation (12) as follows:

(12) $$L\left(n\right)=\sum_{i=0}^{3}\sqrt{{\left(x_{n+1}-x_{n}\right)}^{2}+{\left(y_{n+1}-y_{n}\right)}^{2}+{\left(z_{n+1}-z_{n}\right)}^{2}}$$

The calculation formula of the path energy consumption is expressed in Equation (13) as follows,

(13) $$E\left(n\right)=R1\times \sum_{i=0}^{3}\sqrt{{\left(x_{n+1}-x_{n}\right)}^{2}+{\left(y_{n+1}-y_{n}\right)}^{2}}+R2\times \sum_{i=0}^{3}\left|z_{n+1}-z_{n}\right|$$

### 4.2. Comparative Analysis of Path Planning in a Single-Mound Obstacle Space

The size of the 3D spatial environment map of a single mound obstacle is 100 m × 100 m × 100 m. In the center of the map, there is a cone obstacle growing in the center position. The point represents the starting point, the starting point coordinates are (*x* = 0, *y* = 0, *z* = 0), the star represents the target point, and the end point coordinates are (*x* = 100, *y* = 100, *z* = 60). The number of iterations is 100.

**Figure 13:**
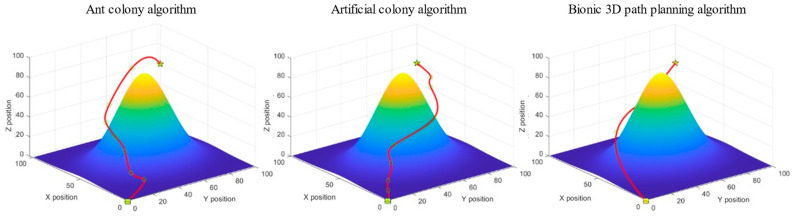
Experimental simulation diagram of a single-mound obstacle space. The box represents the starting point, the pentagram represents the target point, and the middle circle represents the sampling point, in order to smooth the processing path.

The task is to plan an optimal flight path to the target point from the starting position of the UAV below without colliding with the surrounding obstacles. The ant colony algorithm, artificial bee colony algorithm, and bionic 3D path planning algorithm were used to plan the path from the starting point to the target point. The three algorithms were tested in the 3D spatial environment map with obstacles and using the same rules, and the experimental simulation image is shown in Figure 13.

We recorded the path length, iteration duration, and path energy consumption of the three algorithms after 100 iterations, and the experimental results are shown in Table 2. 

**Figure 14:**
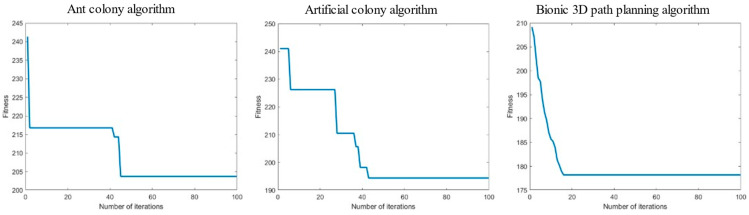
Relationship curve of iteration times—path length in a single-mound obstacle space.

Meanwhile, the relationship curve between the number of iterations and the current calculated path length is provided in Figure 14.

As shown in Table 2, among the path lengths planned by the three algorithms in a single-mound obstacle space environment, the bionic 3D path planning algorithm has the shortest distance, followed by the artificial bee colony algorithm and the ant colony algorithm. The longer the path length, the greater the flight cost for the plant protection UAV to reach the target point. The results show that the bionic 3D path planning algorithm had the lowest flight cost. During the path planning process for a single-mound obstacle space environment, the bionic 3D path planning algorithm had the shortest iteration time, followed by the artificial bee colony algorithm, and the ant colony algorithm had the longest distance. The longer the iteration time, the lower the real-time performance of path planning. The results show that the bionic 3D path planning algorithm had the highest real-time performance. During the path planning process for a single-mound obstacle space environment, the bionic 3D path planning algorithm had the lowest energy consumption, followed by the artificial bee colony algorithm, and the ant colony algorithm had the highest energy consumption. The higher the energy consumption, the less economical the flying of plant protection UAVs. The results show that the bionic 3D path planning algorithm is the most economical. The relationship curve between the number of iterations and path length of the three algorithms in a single-mound obstacle space environment is shown in Figure 14. The bionic 3D path planning algorithm had a better convergence effect than the other algorithms, and its convergence speed and convergence accuracy were better than those of the ant colony algorithm and artificial bee colony algorithm. The faster the convergence rate, the fewer iterations required for path planning, and the faster the optimal path that can be found. The higher the convergence accuracy, the more the actual optimal path agrees with the theoretical optimal path. The results show that the optimal path found by the bionic 3D path planning algorithm is closest to the theoretical optimal path. In summary, the bionic 3D path planning algorithm optimizes the traditional swarm intelligence algorithm and has significant advantages such as a short path length, high real-time algorithm speed, good economy of planned paths, and higher path quality in single-mound obstacle space environments. The bionic 3D path planning algorithm is more suitable in terms of plant protection UAVs operating in single-mound obstacle space environments.

### 4.3. Comparative Analysis of the Path Planning in a Regular Forest Yard Space

The size of the 3D spatial environment map of a regular forest yard is 120 m × 120 m × 120 m. There are nine regular obstacles arranged within the same interval on the map. The point represents the starting point, the starting point coordinates are (*x* = 0, *y* = 0, *z* = 0), the star represents the target point, and the end point coordinates are (*x* = 120, *y* = 120, *z* = 80). The number of iterations is 100.

**Figure 15:**
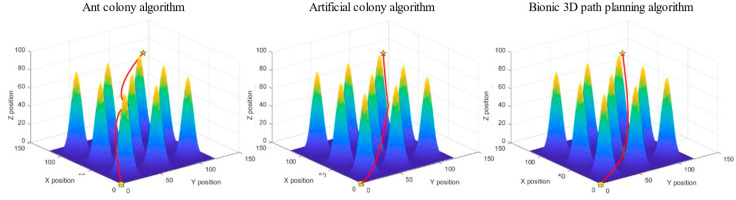
Experimental simulation diagram of regular forest yard space. The box represents the starting point, the pentagram represents the target point, and the middle circle represents the sampling point, in order to smooth the processing path.

The task is to plan an optimal flight path to the target point from the starting position of the UAV below without colliding with the surrounding obstacles. The ant colony algorithm, artificial bee colony algorithm, and bionic 3D path planning algorithm were used to plan the path from the starting point to the target point. The three algorithms were tested in the 3D spatial environment map with obstacles and using the same rules, and the experimental simulation image is shown in Figure 15.

We recorded the path length, iteration duration, and path energy consumption of the three algorithms after 100 iterations, and the experimental results are shown in Table 3. 

**Figure 16:**
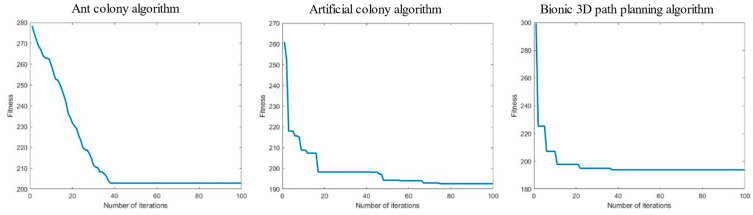
Relationship curve of iteration times—path length in regular forest yard space.

Meanwhile, the relationship curve between the number of iterations and the current calculated path length is provided in Figure 16.

As shown in Table 3, among the path lengths planned by the three algorithms in a regular forest yard space environment, the bionic 3D path planning algorithm has the shortest distance, followed by the artificial bee colony algorithm and the ant colony algorithm. The longer the path length, the greater the flight cost for the plant protection UAV to reach the target point. The results show that the bionic 3D path planning algorithm has the lowest flight cost. During the path planning process for a regular forest yard space environment, the bionic 3D path planning algorithm has the shortest iteration time, followed by the artificial bee colony algorithm, and the ant colony algorithm has the longest distance. The longer the iteration time, the lower the real-time performance of path planning. The results show that the bionic 3D path planning algorithm had the highest real-time performance. During the path planning process for a regular forest yard space environment, the bionic 3D path planning algorithm had the lowest energy consumption, followed by the artificial bee colony algorithm, and the ant colony algorithm had the highest energy consumption. The higher the energy consumption, the less economical the flying of plant protection UAVs. The results show that the bionic 3D path planning algorithm is the most economical. The relationship curve between the number of iterations and path length of the three algorithms in a regular forest yard space environment is shown in Figure 16. The bionic 3D path planning algorithm had a better convergence effect than the other algorithms, and its convergence speed and convergence accuracy were better than those of the ant colony algorithm and artificial bee colony algorithm. The faster the convergence rate, the fewer iterations required for path planning, and the faster the optimal path that can be found. The higher the convergence accuracy, the more the actual optimal path agrees with the theoretical optimal path. The results show that the optimal path found by the bionic 3D path planning algorithm is closest to the theoretical optimal path. In summary, the bionic 3D path planning algorithm optimizes the traditional swarm intelligence algorithm and has significant advantages such as short path length, high real-time algorithm speed, good economy of planned paths and higher path quality in regular forest yard space environments. The bionic 3D path planning algorithm is more suitable in terms of plant protection UAVs operating in regular forest yard space environments.

### 4.4. Comparative Analysis of Path Planning in Irregular Hill Spaces

The size of the 3D space environment map for irregular hills is 100 m × 100 m × 100 m, and there are a number of irregular obstacles randomly arranged on the map. The point represents the starting point, the starting point coordinates are (*x* = 0, *y* = 0, *z* = 0), the star represents the target point, and the end point coordinates are (*x* = 100, *y* = 100, *z* = 60). The number of iterations is 100.

**Figure 17:**
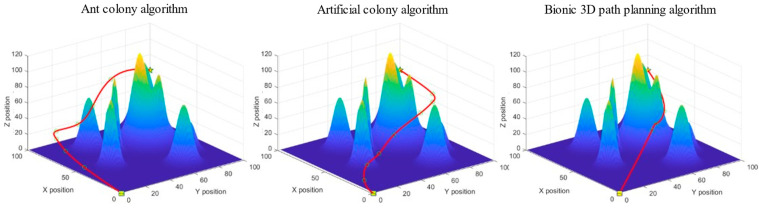
Experimental simulation diagram of irregular hill space. The box represents the starting point, the pentagram represents the target point, and the middle circle represents the sampling point, in order to smooth the processing path.

The task is to plan an optimal flight path to the target point from the starting position of the UAV below without colliding with the surrounding obstacles. The ant colony algorithm, artificial bee colony algorithm, and bionic 3D path planning algorithm were used to plan the path from the starting point to the target point. The three algorithms were tested in the same 3D space environment map of irregular hills, and the experimental simulation image is shown in Figure 17.

We recorded the path length, iteration duration, and path energy consumption of the three algorithms after 100 iterations, and the experimental results are shown in Table 4. 

**Figure 18:**
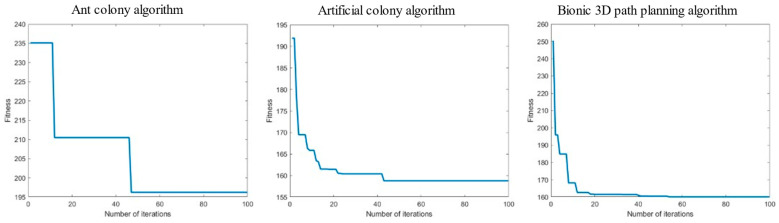
Relationship curve of iteration times—path length in irregular hill space.

Meanwhile, the relationship curve between the number of iterations and the current calculated path length is provided in Figure 18.

As shown in Table 4, among the path lengths planned by the three algorithms in the irregular hill space environment, the shortest distance is the bionic 3D path planning algorithm, followed by the artificial bee colony algorithm, and the longest distance is found by the ant colony algorithm. The results show that the bionic 3D path planning algorithms have the lowest flight cost. During the planning process for the irregular hill space environment, the shortest time of the three algorithms was the bionic 3D path planning algorithm, followed by the artificial bee colony algorithm, and the longest distance was found by the ant colony algorithm. The results show that the bionic 3D path planning algorithms have the highest processing efficiency. During the path planning process for irregular hill space environments, the bionic 3D path planning algorithm had the lowest energy consumption, followed by the artificial bee colony algorithm, and the ant colony algorithm had the highest energy consumption. The results show that the bionic 3D path planning algorithm is the most economical. The curve of the relationship between the number of iterations and the path length of the three algorithms in the irregular hill space environment is shown in Figure 18. The convergence effect of the bionic 3D path planning algorithm is better than that of other algorithms, and the convergence speed and convergence accuracy of the bionic 3D path planning algorithm are better than those of the ant colony algorithm and the artificial bee colony algorithm. The results show that the bionic 3D path planning algorithm needs fewer iterations to find the optimal path. In summary, the bionic 3D path planning algorithm optimizes the traditional swarm intelligence algorithm and has significant advantages such as short, planned path lengths, high real-time algorithm speed, good economy for the planned paths, and a higher quality of paths in the irregular hill space environment. The bionic 3D path planning algorithm is more suitable for plant protection UAVs operating in irregular hill space environments.

### 4.5. Simulation Experiment Conclusions

The comparison of the experimental data of the three algorithms in the space environment of different kinds of obstacles can be seen. In terms of the path length, the bionic 3D path planning algorithm reduced it by 1.6~17.5% compared with the ant colony algorithm, and 1.1~8.7% compared with the artificial bee colony algorithm. In regard to the operation time, the bionic 3D path planning algorithm is 70.21~75.15% shorter than the ant colony algorithm, and 27.56~52.08% shorter than the artificial bee colony algorithm. In terms of path energy consumption, the bionic 3D path planning algorithm reduced it by 17.54~27.35% compared with the ant colony algorithm, and 13.91~18.77% compared with the artificial bee colony algorithm. In terms of the iteration effect, the bionic 3D path planning algorithm achieved the optimal solution with 58~75% less iteration times than the ant colony algorithm and 46~63% less than the artificial bee colony algorithm. The bionic 3D path planning algorithm for plant protection UAVs based on the swarm intelligence algorithm and krill swarm behavior proposed in this paper has a shorter path length, faster operation time, lower path energy consumption, and less iterations. The flight experiments will be conducted in the future to validate the reliability of the bionic 3D path planning algorithm proposed in this paper. Additionally, the algorithm’s implementation may differ across the diverse types of UAVs, and the subsequent step involves experiments, and the analysis of its application and limitations is necessary.

## 5. Conclusions

In this paper, inspired by the movement behavior of krill swarming, a bionic 3D path planning method for plant protection UAVs based on swarm intelligence algorithms and krill swarm behavior was proposed, which improves the problems of the traditional swarm intelligence algorithm such as poor path optimization effect, long running time, and many iterations. In the algorithm design, the adaptive behavior state strategy is introduced, which calculates the optimal fitness of the path in the current environment by constantly switching between the three behaviors according to the current optimization state. In addition to ensuring the convergence rate, the algorithm can also converge to a better precision, which realizes the adaptive global and local convergence of trajectory planning, and improves the convergence speed and accuracy of the algorithm. The optimal flight path is obtained by smoothing the third-order B-spline curve. Finally, the feasibility of the algorithm is verified by experiments in three obstacle space environments, and the bionic 3D path planning algorithm is compared with the traditional ant colony algorithm and artificial bee colony algorithm. The results show that the bionic 3D path planning algorithm is superior to the traditional ant colony algorithm and artificial bee colony algorithm in terms of the length, iteration time, path power consumption, and path quality of the planned path in the three environments. Therefore, the path planning algorithm proposed in this paper can effectively solve problems such as long algorithm operation times and long flight path planning distances, significantly reduce the cost of plant protection UAVs, and efficiently complete the plant protection tasks. At the same time, the research in this paper was a simulation experiment carried out under ideal conditions with no interfering factors. In subsequent research, to carry out experimental verification in a more complex environment, it will be necessary to carry out a progressive study in an environment with multiple interfering factors and dynamic obstacles.

## Figures and Tables

**Table 1:** Body performance constraints.

Parameter	Value
Height above the ground *H_v_*/m	3
Horizontal distance *H_l_*/cm	20
Vertical motion energy consumption *R*_1_(J/m)	120
Horizontal motion energy consumption *R*_2_(J/m)	100

**Table 2:** The experimental results in a single-mound obstacle space.

Algorithm	Lengths/m	Times/s	Energy/J
Ant colony algorithm	161.6551	97.29	1784.02
Artificial colony algorithm	153.5562	82.51	1653.80
Bionic 3D path planning algorithm	150.6818	24.17	1475.18

**Table 3:** The experimental results in regular forest yard space.

Algorithm	Lengths/m	Times/s	Energy/J
Ant colony algorithm	194.4623	119.19	2784.70
Artificial colony algorithm	192.2746	47.67	2753.36
Bionic 3D path planning algorithm	190.3142	35.75	2725.30

**Table 4:** The experimental results in irregular hill spaces.

Algorithm	Lengths/m	Times/s	Energy/J
Ant colony algorithm	216.0701	148.99	3094.12
Artificial colony algorithm	194.8418	59.52	2790.13
Bionic 3D path planning algorithm	178.2282	44.67	2265.82

## Data Availability

Data are contained within the article.
